# Balloon Pulmonary Angioplasty for the Treatment of Chronic Thromboembolic Pulmonary Hypertension

**DOI:** 10.14797/mdcvj.1347

**Published:** 2024-05-16

**Authors:** Lauren N. Carlozzi, C. Huie Lin, Zachary L. Steinberg

**Affiliations:** 1 Seattle Children’s Hospital, Seattle, Washington, US; 2 Houston Methodist DeBakey Heart & Vascular Center, Houston Methodist Hospital, Houston, Texas, US; 3 University of Washington, Seattle, Washington, US

**Keywords:** balloon pulmonary angioplasty, chronic thromboembolic pulmonary hypertension, pulmonary hypertension, pulmonary thromboendarterectomy

## Abstract

Chronic thromboembolic pulmonary hypertension is a rare form of pulmonary hypertension in patients who have evidence of chronic thromboembolic occlusion of the pulmonary vasculature. Historically, surgical pulmonary thromboendarterectomy has been the treatment of choice. However, with up to 40% of patients deemed inoperable, balloon pulmonary angioplasty has emerged as an additional treatment strategy. Balloon pulmonary angioplasty is a complementary strategy alongside surgical pulmonary thromboendarterectomy and offers the opportunity for pulmonary revascularization in patients who have more distal disease, higher comorbidities, or residual obstruction following operative intervention. This review examines the history of balloon pulmonary angioplasty, highlights its effectiveness, discusses important complications and risk reduction strategies, and emphasizes the importance of centers forming a multidisciplinary team of providers to manage the complexity of patients with chronic thromboembolic pulmonary hypertension.

## Introduction

Chronic thromboembolic pulmonary hypertension (CTEPH) is a rare form of pulmonary hypertension in patients who have thromboembolic occlusion of the pulmonary vasculature for more than 3 months.^1^ While a diagnosis of CTEPH mandates the presence of pulmonary hypertension, one subset of symptomatic patients who have normal resting pulmonary artery pressures in the presence of chronic thromboembolic occlusions are classified as having chronic thromboembolic pulmonary disease (CTEPD). It is generally agreed that chronic thromboembolism results from incomplete resorption of acute thrombus following deep venous thrombosis and acute pulmonary embolism (PE). In the absence of early clot clearance, the platelet and red blood cell rich acute thrombotic plug undergoes extensive fibrosis, resulting in a chronic fibrotic plug that is unresponsive to anticoagulation.^2^ The exact pathogenesis of CTEPH/CTEPD is unclear, but risk factors for its development include unprovoked PE, delayed initiation of anticoagulation in acute PE, an underlying hypercoagulable state, diabetes, and hypothyroidism.^3^

Left untreated, CTEPH can lead to pulmonary arterial hypertension of the unobstructed vasculature in the presence of chronic pressure overload, worsening right heart failure, and death.^4^ Ultimately, the treatment for this obstruction is a mechanical solution, and surgical pulmonary thromboendarterectomy (PTE) has long been the treatment of choice in eligible patients. However, up to 40% of patients with CTEPH are deemed inoperable, largely due to either distal lesion location or substantial operative comorbidities.^5,6^ Over the past decade, balloon pulmonary angioplasty (BPA) has emerged as a complementary treatment for CTEPH/CTEPD, utilizing wire and balloon technology created for coronary artery and peripheral arterial vascular interventions. Despite the inability to physically extract a fibrotic plug, BPA has proven an effective means of establishing distal perfusion via sequential balloon dilation of obstructed vessels, leading to a reduction in pulmonary artery pressures and durable improvements in right ventricular function and quality of life.

## History of Balloon Pulmonary Angioplasty

Balloon pulmonary angioplasty for the treatment of CTEPH was first described in 2001 in a landmark report by Feinstein et al. discussing the outcomes of 18 patients with severe PH.^7^ Despite a relatively small number of intervened-upon lesions per patient, significant improvements in hemodynamics and functional class were observed in nearly all patients. However, this came at the expense of a 22% incidence of complications, including three patients who required mechanical ventilation due to pulmonary reperfusion injury and one death from right ventricular failure. The incidence and severity of complications in this initial report overshadowed the beneficial effects of the procedure, leading to a significant lag in the widespread adoption of this treatment modality.

With the publication of a second landmark report in 2012, BPA for CTEPH was recognized as a viable alternative to medical management in nonoperative patients. Mizoguchi et al. reported on the outcomes of 68 patients with CTEPH who underwent repeated BPA and achieved substantial hemodynamic and functional improvements sustained through the 2-year follow-up.^8^ Furthermore, the reported complication rate of 9% was substantially lower than prior publications. In order to optimize safety, patients were empirically treated with inhaled pulmonary vasodilators, inotropic agents, and steroids periprocedurally. Over the subsequent 10 years, BPA for the treatment of CTEPH spread from Japan, across Europe, and into North America and Oceana. With growing experience and expertise, a multitude of centers have reported sustained hemodynamic and functional improvements, a steady decrease in overall complications, and refinements in periprocedural optimization.

## BPA Indications and Patient Selection

While PTE continues to be the standard treatment recommendation for CTEPH, it is estimated that upwards of 40% of CTEPH patients are deemed inoperable.^6,9^ This may be due to operatively inaccessible disease, typically located at or distal to the segmental level, significant operative risk due to medical comorbidities, or residual obstructive disease following initial PTE.^10^ However, because of the substantial benefits of PTE and because centers vary on what constitutes inoperable lesions and prohibitive surgical risk, decisions regarding treatment strategy should be made in multidisciplinary fashion to individualize patient treatment plans.^11,12,13,14^ A thorough review of clinical history, imaging, and functional studies are of paramount importance prior to invasive treatment to ensure the correct diagnosis has been made, risk factors have been identified and addressed, and the entire treatment team is familiarized with each patient to optimize postprocedural care in the event of complications.

All symptomatic patients who are deemed inoperable should be considered for BPA. While there are no recognized absolute contraindications to transcatheter intervention, recognition of multiple patient-specific factors ahead of intervention will help maximize clinical success and decrease serious complications. A recently published consensus statement from the European Society of Cardiology outlines several of these factors, including symptom burden, comorbid conditions, severity of baseline hemodynamics, and identification of high-risk populations such as those who have previously undergone PTE.^15^ Patient education prior to committing to the BPA treatment pathway is of additional importance. Repeated interventions spanning months are often necessary to achieve an optimal result, placing added burden on patients with regard to repeated travel, medical leave, and the need for a strong social support system.

## BPA Outcomes

A multitude of single and multicenter reports on BPA outcomes have appeared over the past decade, initially originating from Japan, with European countries and North America following suit. In general, BPA has proven to result in significant hemodynamic and functional improvements in the vast majority of treated patients across continents. Among the largest of the published multicenter experiences, Ogawa et al. reported the results of 308 patients who underwent a total of 1,408 procedures over 3 years of follow-up.^17^ They observed significant functional improvements as well as an average pre- to postintervention decrease in mean pulmonary pressures (mPAP) from 43.2 to 22.5 mm Hg and pulmonary vascular resistance (PVR) from 853.7 to 359.5 dyne/cm/s^-5^, with an additional decrease in PVR to 288.1 dyne/cm/s^-5^ through the follow-up period. Summing up the combined efforts of many single centers, as well as Ogawa et al.’s multicentered report, Kennedy et al. published a recent meta-analysis including 40 published case series detailing the outcomes of 1,763 patients. Substantial improvements in invasive hemodynamic parameters were observed, with a mean reduction in mPAP by 13.2 mm Hg (95% CI, 14.7-11.8, *P* < .001), PVR by 311 dyne/cm/s^-5^ (95% CI, 350-271, *P* < .001), right atrial pressures by 2.2 mm Hg (95% CI, 2.8-1.6, *P* < .001), and an increase in mean cardiac index by 0.26 L/min/m^2^ (95% CI, 0.17-0.35, *P* < .001). Measures of functional improvement substantially improved as well, with a mean increase in New York Heart Association (NYHA) functional class by 0.9 (95% CI, 1.0-0.8, *P* < .001), World Health Organization (WHO) functional class by 1.0 (95% CI, 1.2-0.9, *P* < .001), and 6-minute walk distance (6MWD) by 70 meters (95% CI, 58-82, *P* < .001).^16^

Smaller single-center reports have evaluated the effects of BPA on ventricular parameters. Fukui et al. observed an improvement in cardiac magnetic resonance imaging (CMR) derived indexed right ventricular end-diastolic volumes from 130 mL/m^2^ to 92 mL/m^2^ (*P* < .001), indexed right ventricular end-systolic volumes from 89 mL/m^2^ to 55 mL/m^2^ (*P* < .001), and right ventricular ejection fraction from 34% to 41% (*P* < .001) in 20 patients who underwent BPA.^18^ Sato et al. demonstrated similar improvements in CMR-derived right ventricular function post-BPA as well as increases in indexed left ventricular end diastolic volumes from 72.1 mL/m^2^ to 86.1 mL/m^2^ (*P* < .01) and indexed left ventricular stroke volume from 41.0 mL/m^2^ to 47.8 mL/m^2^ (*P* < .01).^19^

## BPA Complications

Complications from BPA widely vary from clinically insignificant to life threatening and include vessel perforation, dissection, complete vascular disruption, pulmonary reperfusion injury, and acute right ventricular failure.^10^ Some challenges exist in differentiating between the various types of vascular injury, but prompt recognition is of paramount importance as therapeutic intervention hinges on the appropriate diagnosis, and sparse data is available to help operators with clinical decision-making. In the authors’ opinion, with the exception of pulmonary arterial dissection (which rarely leads to clinical decompensation), vascular injury—such as device perforation (wire, microcatheter, balloon, etc.) or complete vascular disruption—typically presents as hemoptysis within seconds to minutes of the injury and is clinically evident intraprocedurally. While not always possible to identify the exact location of injury by angiography, proximal balloon tamponade of the affected segment usually halts further hemoptysis and allows time for spontaneous healing, reversal of anticoagulation, or device occlusion, as deemed necessary based on the severity of the injury.

In contrast, pulmonary reperfusion injury is more likely to present on the order of hours, typically postprocedurally. Hemoptysis may be a component of the presentation, but often frank hemorrhage is not observed. The hallmark of significant reperfusion edema is worsening hypoxia, which may result in worsening right ventricular dysfunction and death. At present, optimal treatment for reperfusion injury revolves around supportive care that includes supplemental oxygen, mechanical ventilation, inotropic support, and extracorporeal membrane oxygenation.

The meta-analysis by Kennedy et al. reported on complications from 31 individual case series including 6,728 interventions and observed rates of diffuse pulmonary injury of 8.2%, hemoptysis of 7.1%, vessel injury in the absence of hemorrhage or hemoptysis of 5.1%, and 30-day periprocedural mortality of 0.3%.^16^ These results compare favorably to initial single-center outcomes in the early collective experience; however, comparing complication rates between observational studies should be interpreted with caution. Variable definitions of what constitutes a complication, adjudication between vascular and reperfusion injury, patient selection, lesion selection, and operator experience all influence reported rates of complications.

## BPA Risk Reduction

Whenever possible, BPA should be performed by highly trained and experienced interventionalists working in a hospital system that is familiar with CTEPH patients given the disease-specific management issues in this fragile population. It is well recognized that experienced BPA operators may come from a multitude of training pathways, including interventional cardiology, interventional radiology, and cardiothoracic surgery. Given the relatively nascent expertise and limited population requiring BPA, most experts agree that volume is of primary importance to achieve high-quality results and limit complications.

Brenot et al. highlighted the steep learning curve of the procedure, reporting a substantial reduction in complication rates between the first 21 months of the initiation of their center’s BPA program compared to the subsequent 21 months.^20^ Out of 1,006 BPA sessions, there were a total of 113 complications, with a complication rate of 15.8% in the initial period decreasing to 7.7% in the latter half. The most striking complication reduction was seen in patients with severe lung injury, which decreased from 10.4% to 1.8%. Similarly, Mizoguchi et al. demonstrated a reduction in complications between their first 128 procedures and their subsequent 127 procedures, from 35.9% to 23.6%.^8^ The exact number of procedures required to achieve technical proficiency and the number of yearly procedures required to maintain competency remains a matter of debate.

Multiple risk-reduction strategies for BPA have been described. Among the most important, pretreatment with pulmonary vasodilators has repeatedly been shown to reduce intra- and immediate postprocedural complications. In a prospective observational study published by Wiedenrorth et al., pre-BPA treatment with riociguat was associated with an absolute reduction in mortality of 15.4% at 1 year and 31.5% at 5 years compared to a matched cohort of patients who underwent BPA without pulmonary vasodilator pretreatment.^21^ Similar results were observed in the RACE (balloon pulmonary angioplasty versus riociguat for the treatment of inoperable chronic thromboembolic pulmonary hypertension) trial in which a subsegment of patients who received riociguat pretreatment experienced a 28% absolute reduction in serious adverse events at 6 months compared with patients receiving no such pretreatment.^22^

A number of intraprocedural strategies for limiting complications also have been described. Some operators have used pressure-wire-guided BPA in an effort to perform controlled lesion dilations in high-risk patients prone to reperfusion injury, resulting in significant reductions in severe reperfusion edema.^23,24^ A variety of wiring techniques have been employed to reduce distal vascular injury, such as wire knuckling to avoid entrance into tiny branches, the use of nonhydrophilic-coated wires in the very distal vasculature, and the use of microcatheter angiography to ensure an intravascular position.^20,25^ Procedural staging is another important consideration during the first few BPA interventions. Limiting interventions to one or two segmental territories may help to reduce the impact of reperfusion edema in high-risk patients.^7,8^

## Ongoing Topics of Debate

### Anatomic Subtypes and Chronic Total Occlusion Revascularization

**Figure 1:**
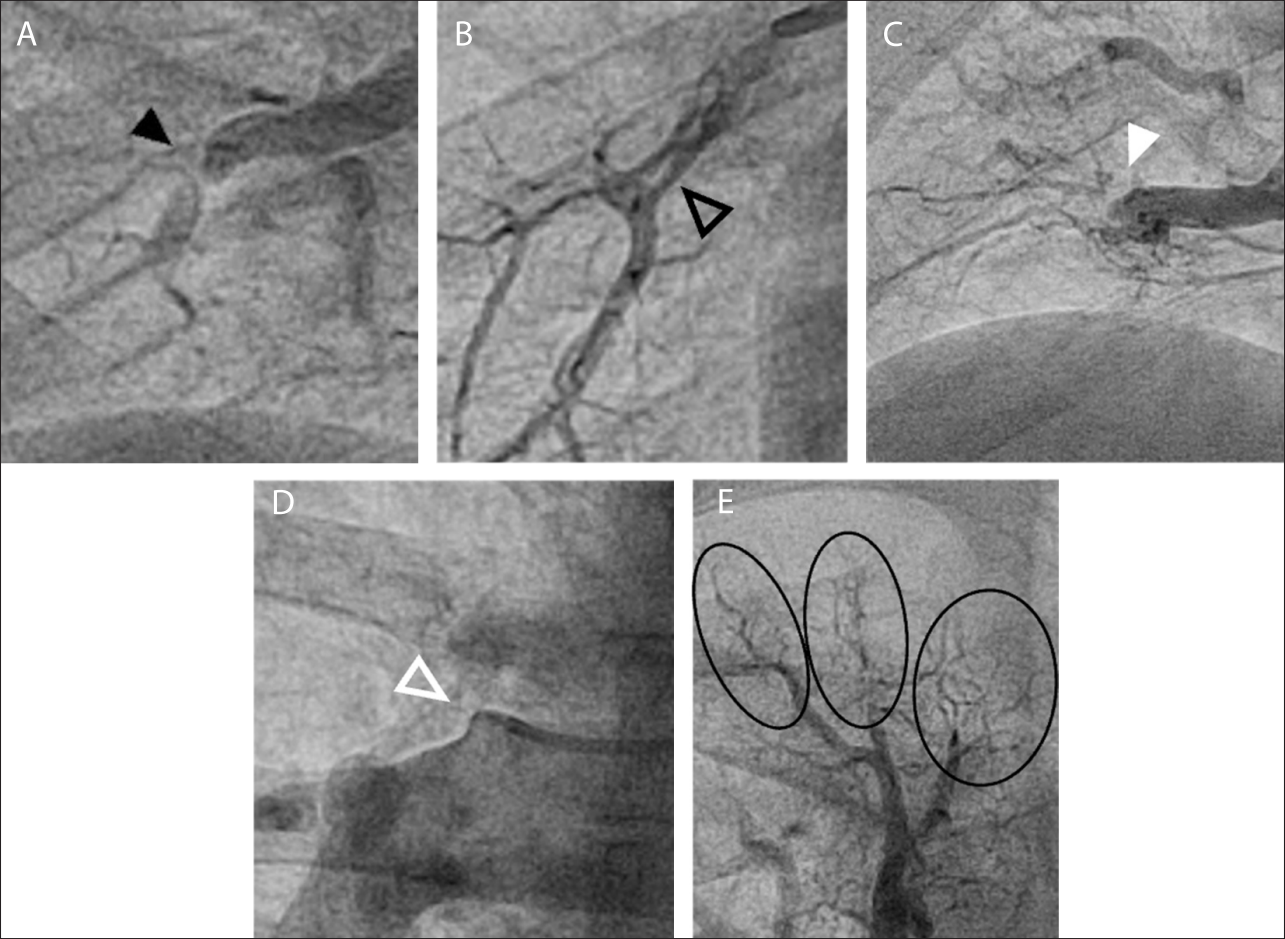
Angiographic depictions of the different chronic thromboembolic lesion subtypes: **(A)** ring lesion (black arrow); **(B)** web lesion (black outlined arrow); **(C)** subtotal occlusion lesion (white arrow); **(D)** total occlusion lesion (white outlined arrow); and **(E)** tortuous lesions (black circles).

As first reported by Kawakami et al. in 2016, five different chronic thromboembolic lesion subtypes, identified by selective angiography, are recognized. These include ring-like stenoses (type A), web lesions (type B), subtotal occlusions (type C), total occlusions (type D), and tortuous lesion (type E) (Figure 1).^26^ Web and ring lesions predominate and are associated with the highest success rates and lowest complication rates. Subtotal occlusions and chronic total occlusions (CTOs) are found less frequently, are associated with lower rates of success, and have higher rates of complications. Considerable debate exists regarding the risk-benefit relationship of revascularizing occluded lesions, especially CTOs. Highly fibrotic proximal occlusion caps, lack of distal visualization, ambiguity of vessel course, and unclear distal vessel pathology have all contributed to this debate. Two of the largest cohorts published to date, Kawakami et al. and Gerges et al., reported on outcomes from interventions on 67 and 352 CTOs respectively.^26,27^ Both centers reported successful vessel revascularization at a rate of approximately 50% and a complication rate of approximately 6%. While others have reported much higher rates of complications with CTO interventions,^28^ it is largely the lower rates of success, specialized equipment, significant time commitment involved in each CTO revascularization, and unclear benefits of CTO revascularization that have sparked debate regarding the value of CTO intervention. However, Gerges et al. observed substantial additive hemodynamic benefits in patients who underwent successful CTO revascularization, with a dose-dependent relationship in hemodynamic improvements based on the number of CTOs revascularized.^27^ While this remains a single-center study, these findings suggest value is added in tackling these complex lesions. A few smaller studies focusing on refining CTO revascularization techniques have reported higher rates of success for CTO revascularization,^25,29^ though easily reproducible techniques and results have yet to be demonstrated.

Intervention on the final lesion group, type E, is often avoided by many operators. Initially described as very tortuous distal vessel pruning, it remains entirely unclear whether these lesions truly represent pathology from tiny emboli versus peripheral arterial vascular disease from another process. In the presence of more macroscopic chronic thromboembolic disease, distal vasculopathy may be a compensatory arteriolar response of the unobstructed vessels to chronically elevated pulmonary pressures. When these lesions occur in isolation, additional workup for diseases that mimic CTEPH, such as a vasculitis, should be considered. Whatever the underlying pathology, interventions on type E lesions carry a low success rate and the highest complication rate.^26^

### Balloon Pulmonary Angioplasty for Chronic Thromboembolic Pulmonary Disease

The safety and efficacy of BPA in patients with CTEPD is less studied than in patients with CTEPH. Patients with CTEPD typically lack the overt resting hemodynamic derangements seen in CTEPH; however, many report functional impairments. Indeed, a growing body of evidence suggests that invasive hemodynamic, magnetic resonance imaging, and cardiopulmonary exercise testing in this patient population can unmask significant hemodynamic abnormalities and exertional limitations,^30,31,32^ which improve following PTE.^31,32^ Furthermore, a few small case series on BPA in patients with CTEPD have demonstrated incremental improvements in both resting and exercise hemodynamics as well as 6MWD and functional class.^28,33^ No significant complications were observed in either cohort. While larger studies investigating the efficacy and safety of BPA in CTEPD are needed, the limited available data supports BPA as a reasonable treatment strategy for patients with exertional symptoms and evidence of chronic thromboembolic obstruction in the absence of resting pulmonary hypertension. There is more clinical equipoise for patients with CTEPD whose symptoms fully respond to pulmonary vasodilator therapy.

## Conclusion

BPA is a highly effective treatment for CTEPH patients, with a substantial and growing body of data demonstrating durable improvements in hemodynamics, ventricular function, exertional capacity, and quality of life. BPA should be viewed as a complementary strategy alongside PTE, offering pulmonary revascularization options for patients with more distal disease, higher comorbidities, and those who have residual obstruction following operative intervention. Procedural safety remains a concern, and multiple techniques to reduce periprocedural complications have been described. Of paramount importance, a multidisciplinary team of providers capable of managing CTEPH patients throughout all phases of treatment should be in place prior to establishing a BPA program to optimize patient outcomes. Many unanswered questions regarding optimal patient selection, interventional technique, and management of complications remain. Close collaboration between BPA centers across continents will undoubtedly speed progress in this regard.

## Key Points

- Balloon pulmonary angioplasty is an effective strategy for nonoperable patients with chronic thromboembolic pulmonary hypertension.
- Balloon pulmonary angioplasty (BPA) can improve both hemodynamic and clinical parameters.
- Candidates for BPA should be evaluated by a multidisciplinary committee to optimize safety and clinical efficacy.
- Ongoing collaboration between centers, prospective data bases, and randomized trials are crucial to advancing progress in this field.
